# Diagnosis and Management of Uterine Rupture in the Third Trimester of Pregnancy: A Case Series and Literature Review

**DOI:** 10.7759/cureus.39861

**Published:** 2023-06-02

**Authors:** Mrooj M Abdulmane, Omar M Sheikhali, Raghad M Alhowaidi, Afshan Qazi, Khalid Ghazi

**Affiliations:** 1 Obstetrics and Gynecology, King Fahad Armed Forces Hospital, Jeddah, SAU; 2 Obstetrics and Gynecology, Ibn Sina National College, Jeddah, SAU; 3 Obstetrics and Gynecology, King Abdulaziz University Faculty of Medicine, Jeddah, SAU

**Keywords:** maternal morbidity, fetal bradycardia, vaginal birth after caesarean section, previous cesarean section, uterine rupture

## Abstract

Background: Uterine rupture is associated with clinically significant uterine bleeding, fetal distress, expulsion or protrusion of the fetus, placenta or both into the abdominal cavity requiring prompt cesarean delivery and uterine repair or hysterectomy. Previous cesarean section is the most common risk factor. The most consistent early indicator of it is the onset of prolonged and profound fetal bradycardia.

Objective: In this study, we present six cases of uterine rupture highlighting risk factors, and challenges in diagnosis and management, along with a review of the literature.

Method: A retrospective case series identified eight cases during the five-year study period. All cases from January 1, 2018 to December 31, 2022 were reviewed. Cases with multiple previous cesarean sections were excluded.

Result: Six cases meeting the study criteria were included in our case series. Uterine rupture was a rare occurrence with a prevalence of nine in 31,315 births representing 0.03% of deliveries. No maternal mortality or need for hysterectomy occurred in our study. Fifty percent of uterine ruptures were associated with stillbirths. The most common risk factor was a previous cesarean section in 83.3%. The most common presenting sign was non-reassuring fetal status patterns in 66.6%. A single case had a silent rupture.

Conclusion: Signs and symptoms of uterine rupture are nonspecific making diagnosis challenging. Delay in definitive management causes significant fetal morbidity and mortality. For best outcomes, vaginal birth after a previous cesarean section needs close monitoring in appropriately prepared units with the ability to perform immediate cesarean delivery and provide advanced neonatal support.

## Introduction

Uterine rupture (UR) is an exceedingly rare and dreaded event that may result in extensive uterine damage requiring a hysterectomy and may even lead to fetal or maternal death [1-3]. It is reported to occur in 1:2,500 to 1:5,000 births, although variations have been reported over time and between different demographic groups [2,4-6]. Two types of UR exist, the less common complete type which results in a direct connection between the uterine cavity and the peritoneal space due to the involvement of all uterine wall layers, and the more common incomplete type where the uterus remains covered by a portion of visceral peritoneum [7].

UR most commonly arises in the setting of a scarred uterus [7-9]. Studies demonstrate a change in the etiology of UR with decreasing cases occurring as a result of manipulative obstetric procedures and increasing prevalence as a consequence of the increased popularity of cesarean section (CS) deliveries [2,6,10,11]. Over the last few decades, the recommended rate of CS deliveries set by the WHO has been surpassed especially in developed countries [12-14]. Diagnosis of UR should be suspected in the setting of altered fetal heart rate patterns or symptoms of vaginal bleeding, maternal tachycardia, or unusual pain during labor [2,10]. In this case series, we will present six cases of UR that occurred in our hospital and share our experience in diagnosis, management, and outcomes as well as a review of the associated literature.

## Materials and methods

A search of the Obstetrics and Gynecology Department’s morbidity and mortality records in our tertiary care hospital from January 1, 2018 to December 31, 2022 was performed to identify all cases of UR. A total of nine cases were discovered and a review of the medical records was performed maintaining complete confidentiality and animosity. All patients who presented throughout the study duration who had one or no prior CS were included. Patients who had multiple CSs were excluded from the study as per our exclusion criteria.

## Results

Our case series showed UR to be a rare complication of delivery with a prevalence of 0.03% (nine in 31,315 births). No maternal mortality occurred in our study and no hysterectomy was required as a result of UR. 50% of UR were associated with stillbirths. Known risk factors of uterine rupture encountered in our patients included previous CS which was seen in 83.3%, multiparity in 33%, obstructed labor in 33%, induction with prostaglandin in 16.2%, and augmentation with oxytocin in 16.2%. No cases were found to have uterine anomalies and no cases of malpresentation were seen. The most common presenting sign for UR in our study was abnormal or non-reassuring fetal status patterns which were seen in 66.6%. We had a single case that presented with a silent UR.

Case one

A 38-year-old gravida 5, para 4 woman with 39+4 gestational weeks of pregnancy was admitted for delivery. Her medical history was free of any medical ailments, surgical history included one CS less than three years ago at 32 gestational weeks due to antepartum hemorrhage and placenta previa during her first pregnancy. Her second to fourth pregnancies were successfully delivered by vaginal birth after cesarean (VBAC). A physical examination showed stable vital signs, her cervix was found to be 3 centimeters dilated, 70% effaced, and vertex at -3 station. Cardiotocographic monitoring (CTG) revealed a reassuring fetal status pattern. She reported that the pain was transiently alleviated after she received meperidine for the labor pain. Her vital signs remained stable, and the heart rate of the fetus was 140 to 145 beats/minute. Two hours later, her ultrasound indicated probable uterine rupture and a non-viable fetus with a breech presentation which was previously cephalic. Repeat evaluation revealed the absence of abdominal pain or PV bleeding and her cervical OS was found to be closed. Consequently, an immediate emergency CS was performed. Intraoperatively, a large amount of hemoperitoneum was noted before a complete rupture of her previous CS scar was observed, which had extended to the cervix with most of the placenta already expelled from it. A fresh stillbirth with an Apgar score of 0-0 weighing 2.8 kg was delivered. The uterus was conservatively repaired in two layers. The mother had an uneventful recovery postoperatively. This case is extremely unique due to the occurrence of a silent UR.

Case two

A case of spontaneous onset of labor in a 29-year-old gravida 2, para 1 woman with 39+3 gestational weeks of pregnancy, with a history of a previous CS delivery was admitted for elective CS due to uncontrolled blood sugar profile. Her medical history was free of any medical conditions except for gestational diabetes, her surgical history included one CS at term two years ago due to fetal distress during her first pregnancy. Her physical examination showed stable vital signs. Examination revealed that she had gone into spontaneous labor with her cervix being 3 centimeters dilated, 50% effaced, and the vertex at -3 station. CTG revealed a reassuring fetal status pattern. Consequently, after a discussion with the patient she elected to deliver vaginally. Following her evaluation an artificial rupture of membrane (ARM) was performed for augmentation and she progressed smoothly to a fully dilated cervix with vertex at -2 station. Suddenly during labor, her CTG revealed fetal bradycardia. As a result, UR was suspected, and an emergency CS was immediately performed. A UR of the entire previous scar was noted. A live baby girl with an Apgar score of 8-9 weighing 3.2 kg was delivered. The uterus was conservatively repaired, and the mother had an uneventful recovery.

Case three**

A 25-year-old gravida 2, para 1 woman at 35+4 gestational weeks of pregnancy was admitted due to a non-reassuring CTG. Her medical history was free of any medical illnesses and her surgical history included a CS at term due to fetal distress two years prior to presentation. She had presented to our department with false labor pain. Her physical examination showed stable vital signs and vaginal examination showed her cervix to be 1 centimeter dilated, 80% effaced, and the vertex at -3 station. CTG revealed a non-reassuring fetal status pattern. Following her evaluation, controlled ARM was done for augmentation and she progressed smoothly to a fully dilated cervix, zero station with caput. An hour later her CTG recorded atypical variable decelerations that did not recover. Consequently, an emergency CS was decided upon as instrumental delivery was not considered appropriate. Intraoperatively a large amount of hemoperitoneum was noted. A complete rupture of her previous CS scar was observed. A live baby boy with an Apgar score of 6-7 weighing 2.8 kg was delivered. The uterus was conservatively repaired and our patient recovered well with no complications.

Case four**

A 27-year-old woman who was gravida 3, para 1, and abortus 1 presented at 33+1 weeks to our emergency department with reduced fetal movements. As a result, she was admitted for fetal monitoring. Her history revealed that she was medically free and her surgical history included a CS at term due to fetal distress three years before her presentation. Evaluation of our patient’s CTG monitoring revealed a pathological CTG pattern of fetal bradycardia and her physical examination showed stable vital signs. A vaginal examination was foregone as her pathological CTG prompted an emergency CS. Intraoperatively, a complete rupture of her previous CS scar was noted. A fresh stillbirth with an Apgar score of 0-0 and weighing 1.9 kg was delivered. The uterus was conservatively repaired in two layers. Her recovery period went smoothly with no complications.

Case five

A 44-year-old grand multiparous woman at 40 gestational weeks of pregnancy was admitted for fetal surveillance due to her antenatal ultrasound showing high resistance fetal umbilical arterial pulsatility index Doppler, as well as a transverse fetal lie. Obstetrical history showed that she was gravida 9, para 7, and abortion 1. Her medical history was free of any medical ailments except for gestational diabetes, surgical history was free. A physical examination showed stable vital signs. On her second day of admission, the fetus’s presentation became cephalic and as a result, she was planned for induction of labor with a prostaglandin agent. She progressed to 4 centimeters dilated, 50% effaced, and the vertex at -3 station. Following her evaluation an ARM was done for augmentation and her CTG demonstrated a reassuring fetal status pattern. She progressed smoothly to a fully dilated cervix, vertex -1, for more than 1 hour. Consequently, oxytocin augmentation was started, however, no further progress had occurred after 1 hour. Her CTG remained reassuring and emergency CS was performed for second-stage arrest. Intraoperatively, a right anterior uterine wall complete rupture was observed with the baby found in the peritoneal cavity. A fresh stillbirth with an Apgar score of 0-0 and weighing 3.9 kg was delivered. The uterus was repaired conservatively and our patient had an uneventful recovery.

Case six**

A 36-year-old woman in her third pregnancy presented in early labor. Her history showed that she was at 39+2 gestational weeks of pregnancy. She was medically free with a surgical history of one CS at term due to twin pregnancy during her first pregnancy, 10 years ago. Her second pregnancy was successfully delivered by VBAC. Her physical examination showed stable vital signs and vaginal examination showed her cervix to be 2 centimeters dilated, 90% effaced, and the vertex at -2 station. CTG revealed a reassuring fetal status pattern. After her evaluation, she was admitted for vaginal. During her admission, ARM was done for augmentation, and she progressed smoothly to 9 centimeters, -1 station; however, no further progress was noted over the ensuing four hours. Her CTG was still demonstrating a reassuring fetal status pattern. Accordingly, an emergency CS was performed for the failure of labor to progress. Intraoperatively an incomplete rupture of her previous CS scar was observed, and a live baby boy delivered with an Apgar score of 8-9 and weighing 2.9 kg was delivered. Her uterus was repaired conservatively, and she had an uneventful recovery period.

## Discussion

Over the last few decades there has been increasing worldwide popularity and performance of CS [15]. Internationally CS birth rates have seen a four-fold increase in less than 20 years [16,17]. The current rate of CS globally is 21.1% ranging from 5% in sub-Saharan Africa to 42.8% in Latin America [17]. The substantial rise in CS rates has led to an increased number of women at risk of complications secondary to a scarred uterus including UR [9,18,19]. Our center is no different, over the study period from January 1, 2018 to December 31, 2022, we had an average of 6,263 deliveries with 33.7% delivered by CS. Current projections show that by 2030 the worldwide rate for CS delivery will be 28.5% with rates as high as 63.4% in Eastern Asia [17].

There is considerable debate and dilemma concerning vaginal births after lower segment CS, paramount to the debate is the concern over UR [20-23]. Most studies indicate that the majority of URs arising from scarred uterus occur in parturient women who have delivered via CS in the past [2,10]. Our study demonstrated similar findings, 83.3% of UR cases were seen in the presence of a scarred uterus following a CS. It is important for Obstetricians to keep in mind that after performing CS they are exposing women to an increased individual risk of UR or CS hysterectomy [22,24]. We recommend counseling patients adequately regarding future risks of UR following CS and having lower thresholds for suspecting UR in women who have undergone prior CS.

Studies looking at women with previous CS deliveries demonstrated that women with two or more CS deliveries were at a higher risk of UR [25]. Limited studies looking at UR in women with two or more CS deliveries found the incidence to range between 0.9% and 3.7% [25-27]. Our case series also highlights the heightened risk of UR in women with previous CS as all patients had a positive history with the exception of a single case. During our review of UR cases in our center we encountered two cases of UR, one in a patient with two previous CS deliveries and the other with four previous CS deliveries; however, both were excluded as per our study criteria. Other risk factors identified in the literature include allowing a trial of labor after CS, instrumental deliveries, or when excessive induction is conducted [28-32]. All women in our case series were allowed a trial of labor after CS except for Case 5, emphasizing its clear association with UR and the heightened risk carried out in such cases. However, no instrumental deliveries were encountered.

Studies have found that the risk for UR decreases in women who have had a successful VBAC [32,33]. A great degree of caution should be taken when managing a trial of labor in women with a previous uterine scar, especially if labor has failed to progress [34]. Although it is difficult to conclude a solid association with certainty from our case series, 33.3% of patients had UR following VBAC, Case 1 had three successful VBACs before suffering UR, and Case 6 also suffered a UR after VBAC. Despite a history of successful VBAC we strongly recommend that all women with previous CS and a consequent uterine scar be closely monitored with intense vigilance for the possible occurrence of UR and that patients should have a decreased thresh-hold for being converted to delivery by CS. Our recommendation is supported by a study that calculated that when labor dystocia; defined as cervical dilatation lower than the 10th percentile and arrested for more than two hours occurred, performing CS would have helped avoid more than 40% of UR [35]. VBAC is only recommended in select cases [36,37]. Careful selection increases the number of successful VBACs as well as decreases maternal complications [22].

Currently, the most effective way of identifying patients at risk of UR remains clinical evaluation as evidenced by studies looking at lower-segment ultrasonography which failed to demonstrate a superiority to clinical evaluation [38-40]. With the widespread use of electronic fetal monitoring, fetal heart rate abnormalities have become the most common presenting feature of UR [2,10]. Fetal heart rate abnormalities may include variable and or late decelerations which occur before the onset of fetal bradycardia; however, no specific fetal heart rate or uterine activity pattern has been identified to indicate UR [41,42]. Our case series also found the importance of fetal heart rate abnormalities as a presenting sign of UR, with fetal heart abnormalities being the first sign to raise suspicion of UR in 66.6% of cases. We suggest that any woman in the active phase of labor should be on continuous fetal monitoring and should receive extensive clinical evaluation and prompt management at the first sign of abnormal pattern, including the possibility of UR, especially in the setting of VBAC.

Thankfully we had no cases of maternal mortality in our case series, with other studies from developed countries showing similar findings [2,6,34]. However, studies from developing countries reported maternal mortality rates ranging from 1% to 13% [42,43]. We believe that this is due to more advanced medical resources and diagnostic modalities available in developed countries allowing UR to be identified and managed adequately at an earlier stage. All women in our case series had successful repair of UR and none required hysterectomy. This is in contrast to other studies which report a hysterectomy rate following UR raging between 13% and 33% [2,9,34]. Fetal mortality occurred in 50% of our patients. Other studies showed fetal mortality rates ranging between 19% and 60% of cases [2,34]. A systematic review found fetal mortality to be 5% in cases of symptomatic UR [9]. We attribute the variations in rates of hysterectomy and fetal mortality following UR to the evolution of medical services in newer studies compared to older ones, as well as the differences in socio-economic statuses allowing earlier recognition between the various countries where the studies were conducted.

## Conclusions

In this case series, we aim to raise awareness regarding the signs and symptoms of UR, which are typically nonspecific, making diagnosis difficult in the absence of a high index of clinical suspicion. Any delay in definitive therapy may carry significant morbidity and mortality for both the fetus and the mother. The inconsistent signs and the short time window for definitive intervention make UR a challenging event even for the most experienced of Obstetricians. We encourage frequent clinical assessment and continuous fetal monitoring to help identify this rare, but often catastrophic complication of pregnancy after previous CS. For the best outcome, VBAC needs to be appropriately managed in a well-staffed and equipped unit for possible immediate CS delivery and advanced neonatal support.
